# Efficacy of PicoSure Laser in Treating Lichen Planus Pigmentosus and Pigmentary Demarcation Lines in Patients With Skin of Color

**DOI:** 10.1111/jocd.70800

**Published:** 2026-04-26

**Authors:** Kiran Sethi, Mona Sharma, Ankur Singh, Manan Gulati, Priyanka Hemrajani

**Affiliations:** ^1^ Isya Aesthetics New Delhi India; ^2^ Department of Dermatology ESIC PGIMSR Basaidarapur India; ^3^ Department of Pathology ESIC PGIMSR Basaidarapur India

**Keywords:** laser, Lichen Planus Pigmentosus, PicoSure, Pigmentary Demarcation Lines, skin of color

## Abstract

**Background:**

Lichen Planus Pigmentosus (LPP) and Pigmentary Demarcation Lines (PDL) are common pigmentary disorders among individuals with skin of color (SoC), presenting challenges for treatment due to risks of side effects, such as post‐inflammatory hyperpigmentation (PIH). PicoSure laser, a 755‐nm picosecond laser, has shown promise in treating pigmentary conditions with minimal adverse effects.

**Aims:**

To evaluate the safety and efficacy of PicoSure laser treatment in patients with LPP and PDL, focusing on pigmentation reduction, patient satisfaction, and adverse effects.

**Patients/Methods:**

A prospective case series was conducted over 16 weeks, involving 10 patients (5 LPP, 5 PDL) with Fitzpatrick skin types III–V. Patients underwent PicoSure laser treatment using a 755‐nm wavelength, with assessments based on pigmentation reduction, patient satisfaction (via a visual analogue scale), and adverse effects.

**Results:**

All patients demonstrated significant improvement in pigmentation. In LPP cases, 4 patients achieved > 90% improvement (Grade V), while 1 patient showed > 75% (Grade IV) improvement. PDL patients had > 75% (Grade IV) improvement in the majority of the cases. No significant adverse effects were reported, with minimal PIH observed. High patient satisfaction was reported across all cases.

**Conclusions:**

PicoSure laser offers a safe and effective treatment for LPP and PDL in patients with skin of color, providing significant pigmentation reduction with minimal risk of PIH. It serves as a promising treatment alternative to traditional lasers, with potential for broader application in this demographic. Further studies with larger sample sizes are warranted to confirm long‐term efficacy.

## 1 Introduction

The field of ethnic dermatology has gained prominence as the global population of individuals with skin of color (SoC) continues to grow. Although SoC benefits from the protective effects of melanin against skin cancer, it is more prone to multiple pigmentation disorders, such as Lichen Planus Pigmentosus (LPP) and darker Pigmentary Demarcation Lines (PDL). These conditions can cause significant psychological and social distress, especially when they affect visible areas. Managing conditions like LPP and PDL is challenging due to the risk of side effects and the limited effectiveness of conventional treatments. However, advancements in laser therapies offer promising solutions by providing targeted treatment with minimal damage to the surrounding skin [1].

Picosecond lasers, introduced in the 1990s, have shown superior effectiveness in clearing stubborn tattoo pigments compared to Q‐switched (QS) lasers, even in cases unresponsive to multiple QS treatments. For instance, Chesnut et al. reported successful use of a 755‐nm picosecond Alexandrite laser for a resistant nevus of Ota [2]. With their shorter pulse duration and high peak power, picosecond lasers cause less epidermal damage and have a lower risk of post‐inflammatory hyperpigmentation (PIH). This makes them particularly relevant for treating challenging conditions like LPP and PDL in the skin of color population, where minimizing adverse effects is crucial. This study aims to evaluate the safety and efficacy of PicoSure laser treatment for LPP and PDL in patients with skin of color [3, 4].

## 2 Methodology

- Study design: Prospective case series conducted over 16 weeks.
- Patient selection:
- 10 patients with Fitzpatrick skin types III–V.
  ○ 5 patients with LPP and 5 with PDL.
  ○ Diagnosis was based on clinical assessment and dermoscopic findings. Histopathological confirmation was not performed in all cases, as biopsy consent was not obtained from all patients.
- Inclusion criteria: Stable LPP for at least 6 months, presence of PDL, no prior laser treatment.
- Exclusion criteria: Keloid tendency, recent dermatological treatment, active infections, systemic illness, photosensitivity, pregnancy, or lactation.
- Ethical approval was obtained as per institutional norms, and written informed consent was taken from all participants.

### 2.1 Laser Protocol

- Wavelength: 755 nm
- Pulse duration: 750 ps
- Spot size: 6 mm
- Frequency: 5 Hz
- Lens: FOCUS
- Sessions repeated at 30‐day intervals

### 2.2 Evaluation Criteria

- Clinical assessment of pigmentation reduction.
- Patient satisfaction via Visual Analogue Scale (1–5).
- Documentation of adverse effects (e.g., erythema, PIH).

## 3 Results

- PDL patients were all female, with a mean age of onset of 31.8 years and an average disease duration of 3.5 years.
- LPP patients had a mean age of 24.5 years and a disease duration of 4.8 years.
- No associated pigmentary or systemic conditions were observed in either group.

PDL subtypes were classified based on the anatomical location:

- F: Forehead/frontotemporal region
- G: Malar (cheek) region
- H: Mandibular and perioral areas
- Combinations (F + G, F + H, G + H) indicate involvement of multiple zones.

## 4 Discussion

**FIGURE 1:**
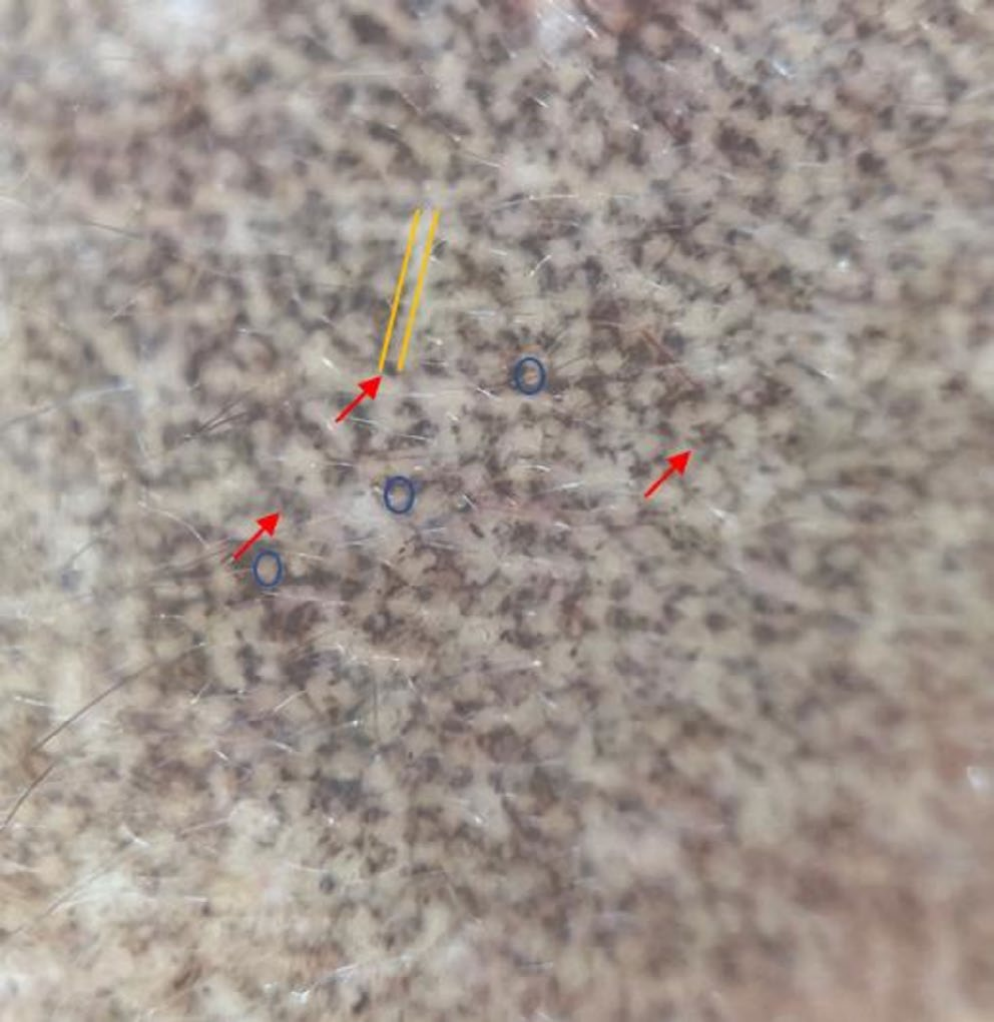
Dermoscopy (10×) shows a light to dark brown background with numerous randomly distributed brown to gray‐brown dots and globules (red arrows), peri‐eccrine dots (blue arrows), and linear annular granular (hem‐like) structures (yellow lines) sparing the skin creases suggestive of Lichen Planus Pigmentosus (LPP).

This prospective case series evaluated the clinical profiles and treatment outcomes of LPP and PDL in patients with Fitzpatrick skin types III–V. The diagnosis of LPP was established based on clinical examination and dermoscopic findings. Patients typically presented with bilateral, slate‐gray macules, without preceding erythema or pruritus. Dermoscopy revealed light to dark brown background with numerous randomly distributed pseudo‐network of brownish‐gray dots and globules indicative of pigment incontinence, peri‐eccrine dots and linear annular granular structures (hem‐like) sparing the skin creases (Figure 1).

PDL presented as well‐defined, symmetrical pigmentation localized to the frontotemporal and malar regions.

The 755‐nm picosecond Alexandrite laser, with its ultrashort pulse duration, is markedly shorter than the thermal relaxation time of melanosomes. This enables selective photothermolysis of melanin with minimal collateral damage to surrounding structures, including hemoglobin‐rich vasculature and the epidermis. In contrast, low‐fluence 1064‐nm Q‐switched Nd:YAG (QSNY) laser treatments for melasma and PIH often plateau in efficacy after multiple sessions. Picosecond lasers, owing to their shorter pulse width and higher peak power, allow more precise melanin fragmentation while minimizing adverse effects. Moreover, the ability to deliver lower fluence levels while maintaining efficacy further reduces the risk of complications. Despite its higher cost, the picosecond laser demonstrates superior efficacy and safety compared to conventional nanosecond Q‐switched lasers. It is particularly beneficial in treating recalcitrant pigmentary disorders such as melasma and PIH in patients with darker skin tones.

In this study, the mean age of onset was 24.5 years for LPP and 31.8 years for PDL, with a female predominance observed in both groups—likely influenced by hormonal factors or cosmetic use. LPP patients reported greater daily sun exposure, suggesting ultraviolet radiation as a contributory factor. The absence of systemic associations or other lichen planus variants in LPP patients indicated isolated facial involvement. Commonly implicated triggers included cosmetic creams, hair dyes, and perfumes (Tables 1 and 2). PDL cases showed characteristic linear and symmetrical pigmentation, predominantly in the frontotemporal and malar regions. Some patients exhibited moderately defined or asymmetrical lesions. No systemic diseases or other pigmentary disorders were identified in PDL patients.

**TABLE 1:** Demographic and clinical details of patients with Pigmentary Demarcation Lines (PDL).

Parameter	
Gender	
Female	5 (100%)
Male	Nil
Mean age of onset	31.8 years (20–40 years)
Mean duration	3.5 years (6 months to 10 years)
Average daily sun exposure	2.8 h
Photoprotection	
Regular use of sunscreen	3 (60%)
Physical barriers of photoprotection	2 (40%)
Family history of facial PDL	0
Regular menstrual cycle	5 (100%)
Use of oral contraceptive pills or hormones	0
Onset of PDL in pregnancy	0
Type of facial PDL	
“F” alone (Forehead and frontotemporal areas)	3 (60%)
“G” alone (Malar region)	1 (20%)
“H” alone (Mandibular and perioral regions)	0
“F” and “G” (Both forehead & cheeks)	1 (20%)
“F” and “H” (Forehead and jawline)	0
“G” and “H” (Cheeks and jawline)	0
Associated pigmentary changes (Periorbital pigmentation/Freckles/Lentigenes/Melasma)	NIL

**TABLE 2:** Demographic and clinical details of patients with Lichen Planus Pigmentosus.

Gender	
Female	5 (100%)
Male	0
Mean age (years) Range	24.5 years (20–45 years)
Mean duration of disease	4.8 years (2 months to 10 years)
Average daily sun exposure	3.5 h
Most commonly involved sites	
Face	5 (100%)
Neck	0
Etiological agents	
Mustard oil/Almond oil	1 (20%)
Hair dye	4 (80%)
Perfumes/deodorants	3 (60%)
Cosmetic creams	2 (40%)
Photoprotection	
Regular use of sunscreen	3 (60%)
Physical barriers of photoprotection	1 (20%)
Family history	Nil
Regular menstrual cycle	5 (100%)
History of drug intake	Nil
Pattern of pigmentation	
Diffuse	5
Reticular	Nil
Associated diseases (Vitiligo/Hypothyroidism/Hepatitis C/Chronic urticaria/Tuberculosis/Canities/Concomitant Lichen planus)	Nil

**FIGURE 2:**
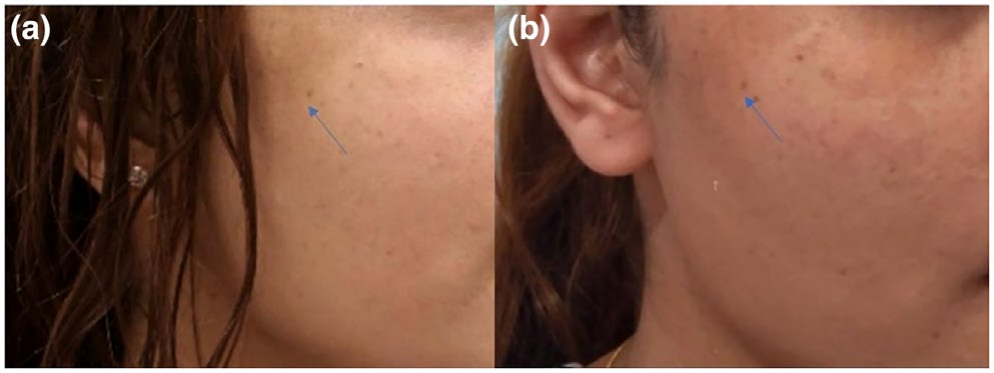
A 28‐year‐old woman with Pigmentary Demarcation Lines. (a) Before treatment (b) After treatment.

**FIGURE 3:**
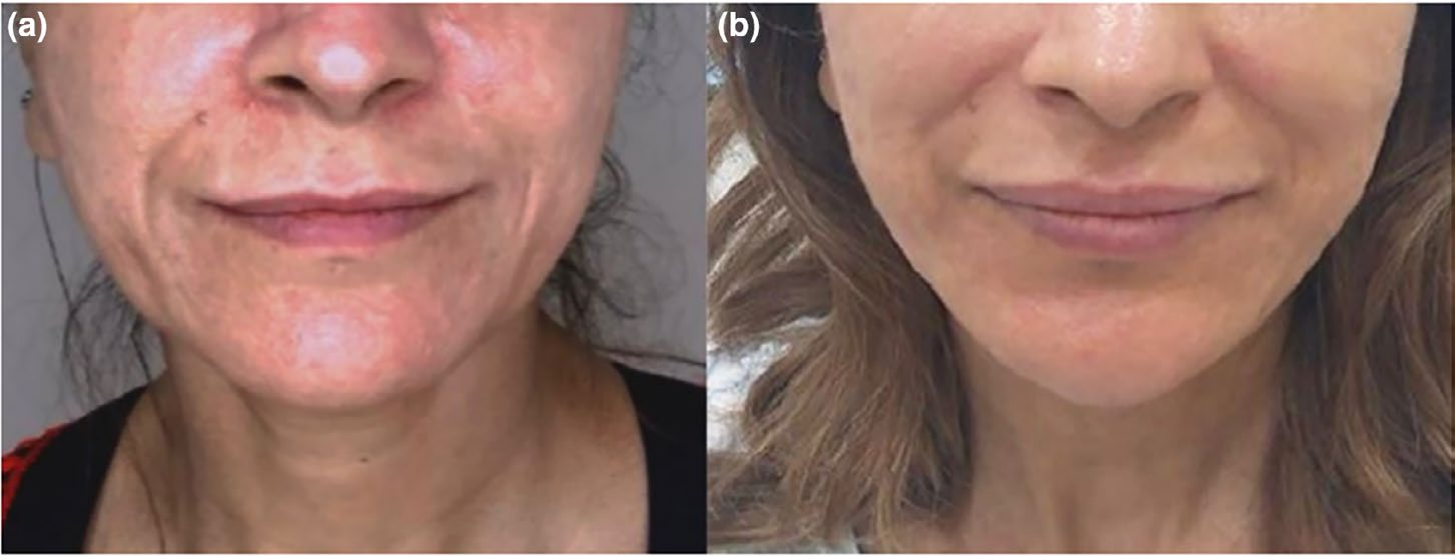
A 38‐year‐old woman with Lichen Planus Pigmentosus. (a) Before treatment (b) After treatment.

All patients underwent four treatment sessions with the 755‐nm picosecond laser using a FOCUS lens, spaced at monthly intervals. Both LPP and PDL responded favorably to treatment. Specifically, 4 LPP patients achieved > 90% improvement (Grade V) improvement, and one achieved ≥ 75% improvement (Grade IV). No adverse effects, such as post‐inflammatory hyperpigmentation (PIH), were observed (Table 3). These results align with prior reports supporting the use of picosecond lasers for challenging hyperpigmentation disorders, reinforcing its safety and efficacy in managing both LPP and PDL (Figures 2 and 3).

**TABLE 3:** Profile of patients and results.

Age	Sex	Color of lesions	Morphology of lesions	Number of sessions	Percentage of improvement	Complications
Lichen Planus Pigmentosus						
20 years	F	Bluish gray	Malar area	4	Grade 5	Nil
27 years	F	Bluish gray	Malar area and temple	4	Grade 5	Nil
23 years	F	Bluish gray	Malar area and temple	4	Grade 5	Nil
30 years	F	Bluish gray	Malar area and temple	4	Grade 5	Nil
45 years	F	Black	Temporal area	4	Grade 4	Nil

Age	Sex	Type of PDL	Morphology of lesions	Number of sessions	Percentage of improvement	Complications
Pigmentary Demarcation Lines (PDL)						
29 years	F	F alone (involving forehead and frontotemporal areas)	Well‐defined and symmetrical	4	Grade 4	Nil
32 years	F	G alone (involving malar region)	Well‐defined and symmetrical	4	Grade 4	Nil
36 years	F	F alone (involving forehead and frontotemporal areas)	Well‐defined and symmetrical	4	Grade 3	Nil
33 years	F	F (involving forehead)	Moderately defined and symmetrical	4	Grade 4	Nil
40 years	F	Both F & G (involving both forehead and cheek regions)	Asymmetrical, moderately defined	4	Grade 4	Nil

*Note:* Grade I: < 25% improvement, minimal improvement. Grade II: 26%–50% improvement, moderate improvement. Grade III: 51%–75% improvement, marked improvement. Grade IV: > 75% improvement, good improvement. Grade V: > 90% improvement, almost clear.

Individuals with skin of color (Fitzpatrick types III–V) are more susceptible to PIH following laser treatments. However, the rapid pulse duration of the Picosure laser significantly reduces thermal injury, thereby lowering this risk. In our study, no significant adverse events or PIH were reported, highlighting the safety of this modality in darker skin types. Patient‐reported outcomes also reflected high satisfaction, affirming the clinical acceptability and utility of this treatment in skin of color populations.

Traditional Q‐switched lasers have been employed in similar cases but typically require more sessions and are associated with a higher incidence of side effects in darker skin. In contrast, the Picosure laser delivers energy in shorter bursts, making it a more efficient and safer option. Existing literature supports the superiority of picosecond lasers over nanosecond lasers in providing faster and more consistent outcomes in pigmentary disorders [5, 6, 7].

Q‐switched Nd:YAG lasers, particularly the 1064‐nm wavelength, have traditionally been used for conditions such as LPP and PDL but often require a higher number of sessions and are associated with a greater risk of post‐inflammatory hyperpigmentation (PIH), especially in Fitzpatrick skin types III–V. Several studies have reported only moderate improvement with Q‐switched lasers in these populations. In contrast, the PicoSure laser, with its picosecond pulse duration and higher peak power, offers more precise melanin targeting and reduces thermal injury, translating into fewer sessions, better outcomes, and lower incidence of adverse effects. Our results, demonstrating ≥ 75% improvement in all cases with no significant PIH, reinforce the superior efficacy and safety profile of picosecond lasers as a preferred modality for treating recalcitrant pigmentation in darker skin tones [8].

Despite the overall success, a few patients with longstanding LPP did not achieve complete clearance, suggesting that disease chronicity and pigment depth may affect treatment efficacy. Additionally, the high cost of Picosure laser therapy may limit accessibility for patients from lower socioeconomic backgrounds. A longer follow‐up period is essential to assess the durability of treatment outcomes, as pigmentation recurrence remains a concern in chronic cases like LPP.

Future research involving larger cohorts and broader skin type representation is necessary to develop standardized protocols for treating LPP and PDL using the Picosure laser. Combining laser therapy with adjunctive treatments, such as topical depigmenting agents, may further improve results and reduce recurrence. Investigating the specific mechanisms by which picosecond lasers modulate melanogenesis could enhance understanding and optimization of treatment strategies for pigmentary disorders in skin of color populations.

## 5 Limitations

This study has several limitations. The small sample size and absence of a control or comparative arm limit the generalizability and strength of the conclusions. Histological confirmation was not performed for all cases, which may affect diagnostic accuracy. The short follow‐up period prevents assessment of long‐term outcomes and recurrence, especially in chronic conditions like LPP. Additionally, all participants were female with Fitzpatrick skin types III–V, limiting applicability to other populations. Further studies with larger, diverse cohorts, histological validation, and extended follow‐up are warranted.

## 6 Conclusion

The Picosure laser shows considerable promise in managing pigmentary disorders such as LPP and PDL in skin of color populations. It offers a safer, more effective alternative to traditional laser therapies, with high patient satisfaction and minimal risk of adverse effects. Its role as a first‐line treatment is particularly valuable for patients unresponsive to conventional modalities.

## Author Contributions

Study concept and design: K.S., M.S., P.H. Analysis and interpretation of data: K.S., M.S., P.H. Drafting of the manuscript: M.S., P.H. Critical revision of the manuscript for important intellectual content: K.S., M.S., A.S., P.H. Statistical analysis: K.S., M.S., M.G., P.H. Obtained funding: None.

## Ethics Statement

This study was conducted in accordance with the Declaration of Helsinki. Institutional ethical committee approval was obtained prior to initiation of the study. Written informed consent was obtained from all participants for both treatment and publication of anonymized clinical data.

## Consent

Written and informed consent from patients taken as per institutional norms. Also patient's identity is not disclosed.

## Conflicts of Interest

The authors declare no conflicts of interest.

## Data Availability

The data that support the findings of this study are available from the corresponding author upon reasonable request.
